# The Mycology of the Female Organs of Generation in Health

**Published:** 1889-05

**Authors:** 


### *THE MYCOLOGY OF THE FEMALE ORGANS OF GENERATION IN HEALTH.*

A SYSTEMATIC investigation has been recently conducted by Dr. G. Winter (*Zeitschrift für Geburtshülfe und Gynäkologie*), in order to determine the nature of the microörganisms that are normally found in the sexual organs of healthy women. The author states that the Fallopian tubes in health contain no microbes of any kind, and this also applies to the cavity of the uterus. In about one-half of the cases examined, the region of the internal os showed the presence of various microbes. The cervical canal is almost invariably the seat of numerous organisms, and it is a noteworthy fact that their number rapidly increases in the condition of pregnancy. In the vagina in healthy women, various microbes are a constant occurrence.

Dr. Winter has also found that there is a decided preponderance of staphylococci over other varieties of germs in the genital organs of women. He assumes that they are identical with the pathogenous cocci, but admits that his inoculation experiments were not conclusive. The explanation given of this apparent inconsistency is plausible enough. Normally these microbes vegetate in a condition of lessened virulence; but if putrescible material or necrotic tissues supplant the healthy structures, their original virulence is fanned into activity. The practical inference is seen at a glance; and it may be stated that most gynecologists are already practising the strict antisepsis which these observations would place in the light of an absolute necessity rather than an optional procedure.—*Editorial in Medical Record.*

---

SPONTANEOUS INVERSION OF THE UTERUS after the placenta left it, was observed by Gustav Braun, in Vienna, twice in the same woman. —*Wiener Klinische Wochenschrift, No. 1, page 8. 1889.*

